# Supplementary material for: Investigation of Virulence-Related Markers in Atypical Strains of Toxoplasma gondii from Brazil
Source: Microorganisms. 2025 Jan 29;13(2):301. doi: 10.3390/microorganisms13020301 (PMC11857696; doi:10.3390/microorganisms13020301)

Supplementary Figure S1: Representative gel images of PCR-RFLP genotyping (markers GRA15, ROP5, ROP16, ROP17 and ROP18). Sample IDs are at the top of the gel images, genotype results are at the bottom. MK is the DNA size marker.

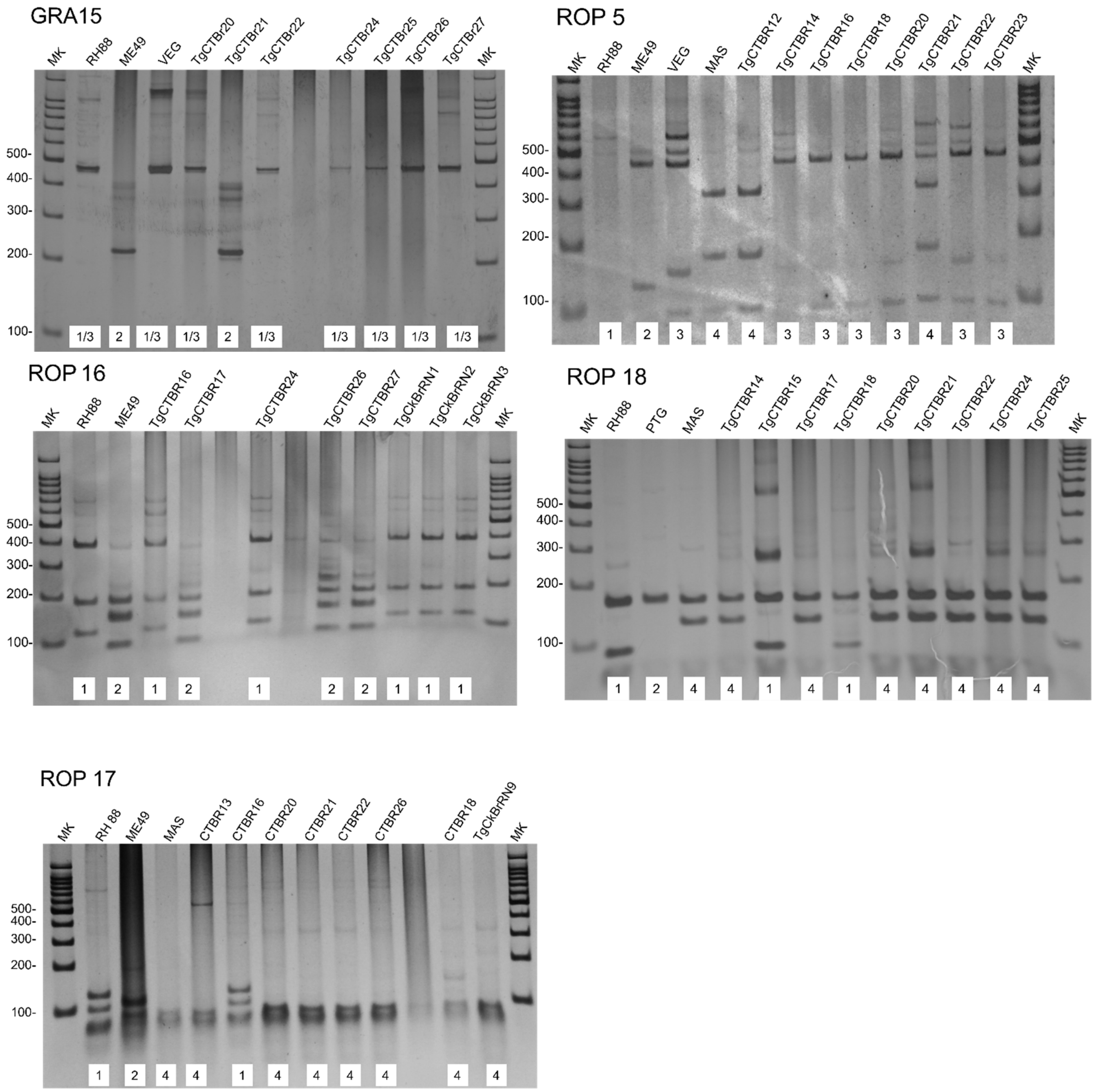

Supplement: Supplementary file 1 [file microorganisms-13-00301-s001.zip › Suplemmentary Figure S1 - 18-12-24.pdf]
